# A case report of rare ectopic pheochromocytoma adjacent to pancreas

**DOI:** 10.1097/MD.0000000000020858

**Published:** 2020-06-19

**Authors:** Chenshan Jiang, Jianguo Zhao, Li Sun, Bing Cai

**Affiliations:** Department of General Surgery, Wuxi People's Hospital Affiliated Nanjing Medical University, Jiangsu Wuxi, China.

**Keywords:** ectopic pheochromocytoma, hypertension, misdiagnosis, pancreatic space-occupying lesion, surgery

## Abstract

**Rationale:**

Ectopic pheochromocytoma is a special type of pheochromocytoma which occurs outside the adrenal gland. The most common symptoms of ectopic pheochromocytoma are palpitations, headaches, profuse sweating, and hypertension. In clinical practice, diagnosis of ectopic pheochromocytoma remains difficult.

**Patient concerns:**

The patient was a 43-year-old female who was admitted to our hospital with the chief complaint of upper abdominal discomfort for 1 week. Computed tomography demonstrated a neoplasm in the head of pancreas. The patient also had history of hypertension and type-2 diabetes.

**Diagnosis:**

According to the postoperative pathological examination, the lesion was mainly composed of chromaffin cells. Immunohistochemical staining revealed that the tumor expressed chromogranin A, NSE, and synaptophysin. Based on these findings, this mass was diagnosed as benign ectopic pheochromocytoma (paraganglioma).

**Interventions:**

Surgical resection operation was carried out and the patient's blood pressure was monitored continuously. Vasopressor or anti-hypertensive drugs were used according to circumstances.

**Outcomes:**

The patient recovered well and was discharged from hospital with normal blood pressure.

**Lessons:**

This report reminds us to pay close attention to the likelihood of ectopic pheochromocytoma and other low-incidence diseases. Physicians and imaging clinicians should explore all clinical possibilities to avoid missed diagnosis or misdiagnosis of ectopic pheochromocytoma and take effective treatment measures to maximize patient benefits.

## 1 Introduction

Pheochromocytoma is a chromaffin cell tumor commonly arising from adrenal medulla. It has been reported the annual incidence is 0.4/1 million to 9.5/1 million.^[1]^ The rule of “tens” is often stated to reflect the distribution and histology of pheochromocytomas, with 10% being bilateral, 10% ectopic in origin, and 10% malignant.^[2]^ Ectopic pheochromocytoma is considered as pheochromocytoma located at extra-adrenal site, which commonly lies near abdominal aorta, followed by bladder, mediastinum, and head.^[3]^ Pheochromocytoma releases a large amount of catecholamine, which causes a series of symptoms commonly presenting with episodes of headaches, sweating, palpitations, and hypertension.^[4]^ Due to its variable clinical symptoms, diagnosis of ectopic pheochromocytoma remains difficult. The etiology of the tumor is not fully understood. Ectopic pheochromocytoma (EP) is not sensitive to radiotherapy or chemotherapy, so surgery remains the mainstay of treatment of all ectopic pheochromocytomas.^[5]^ This case involves a patient with ectopic pheochromocytoma who presented atypical symptoms initially diagnosed as space-occupying lesions of the pancreas. In this article, we examine the clinical features of this case, report the diagnosis and treatment process.

## 2 Case report

The patient was a 43-year-old female who was admitted to our hospital in September 2019 with the chief complaint of upper abdominal discomfort for 1 week. One week prior to admission, the patient developed upper abdominal discomfort. The condition was not related to any obvious cause. She went to a local hospital, where she underwent upper abdominal computed tomography (CT), which yielded the following results: neoplasm in the head of pancreas. On September 14, the patient was transferred to our hospital for further therapy. Her history included hypertension treated for the previous 2 years with a beta-blocker and type-2 diabetes controlled by proper diets and exercises. The patient presented emaciation while no other obvious symptoms like headache, palpitation or arrhythmia were presented.

The patient physical examination showed a height of 165 cm, a weight of 43 kg, a body temperature of 36.6°C, a respiratory rate of 18 breathes/min, a heart rate of 80 beats/min, and blood pressure of 130/80 mm Hg. The patient had clear mind. The skin all over the body and sclera was not icteric, while no hemorrhagic spots or ecchymoses were found. The superficial lymph nodes were not palpable or enlarged. The abdomen was flat with normal abdominal breath. Marked tenderness pain over the middle and upper abdomen was presented with no rebound tenderness or muscular tension. Liver and spleen were impalpable and gurgling sound was normal.

**Figure 1:**
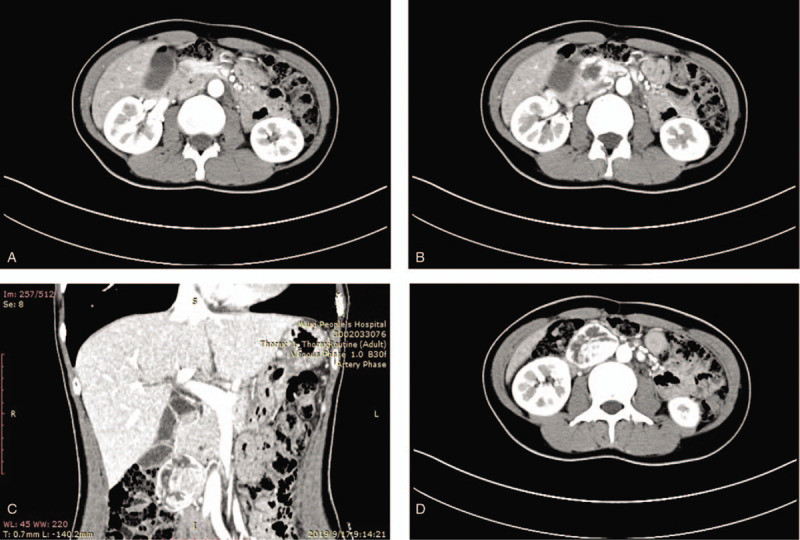
Abdominal computed tomography performed 3 days after admission of the 43-year-old female patient. (A, B, and C) Space-occupying mass lesion below the head of pancreas. (D) The mass had rich blood supply.

After admission, hepatic function examination yielded the following results: alanine transaminase (ALT) 13 U/L, aspartate aminotransferase (AST) 15 U/L, total bilirubin 18.6 μmol/L, direct bilirubin 5.3 μmol/L, and indirect bilirubin 13.3 μmol/L; examination of tumor biomarkers revealed that carcinoembryonic antigen (CEA), carbohydrate antigen125 (CA125) and carbohydrate antigen 199 (CA199) were all in the normal ranges; glycosylated hemoglobin test showed 5.6%,which was normal. Renal function was normal. An abdominal CT scan was performed without, and then with, injection of iodinated contrast agent in the portal phase. It demonstrated that the space-occupying mass lesion which was located below the head of pancreas (Fig. 1A–C) had rich blood supply (Fig. 1D).

Considering the patient's clinical signs including typical upper abdominal pain, type-2 diabetes history, as well as her clinical imaging report, the patient received the preoperative diagnosis as space-occupying lesions of the pancreas, most likely pancreas cancer. Taken the patient's hypertension and emaciation into consideration, the tumor also had a possibility to be pheochromocytoma. Still, surgical excision was considered as the best option.

**Figure 2:**
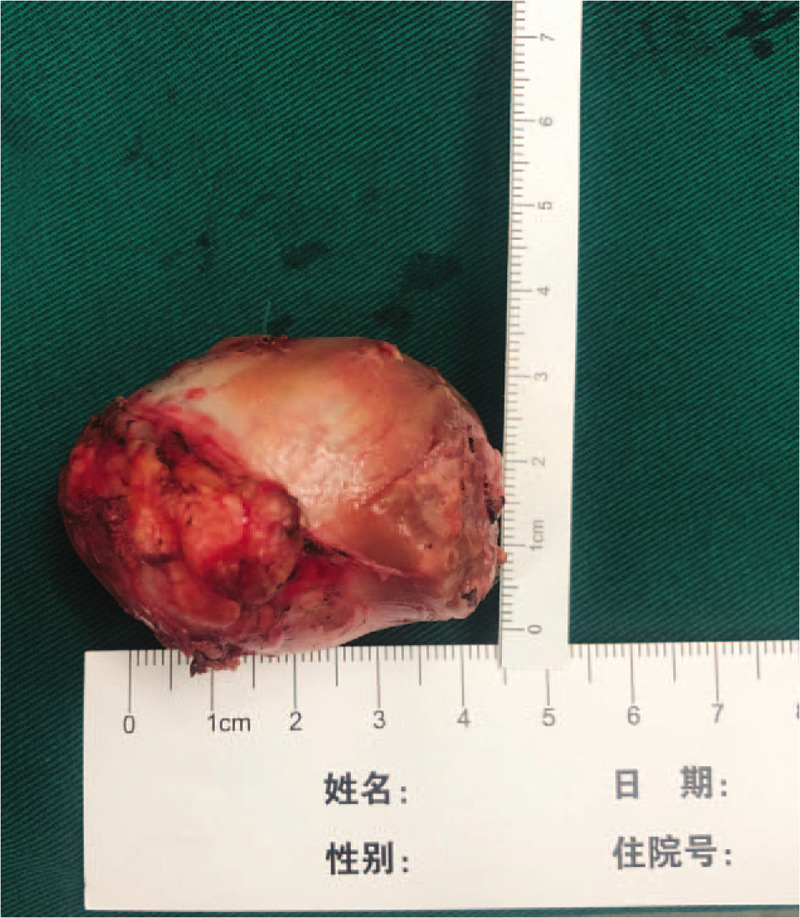
Surgical specimen of the ectopic pheochromocytoma.

After implementing measures to exclude surgical contraindications, the patient received surgery under general anesthesia on September 23. During surgery, we routinely explored the abdominal cavity and found that liver's border was blunt, and no obvious abnormality was observed in stomach, duodenum or transverse colon. An irregular 5∗4 cm hard mass with distinct edge was present below the pancreas, which was adjacent to abdominal aorta and inferior vena cava, also on the right side of mesenteric vessel. During operation, when we touched the mass, the patient presented great undulation of blood pressure, with a maximum value of 200/100 mm Hg. At this point, the patient received an intraoperative diagnosis of ectopic pheochromocytoma. After informing the patient's family members of the intraoperative findings, the details of the possible adverse prognosis, they signed the informed consent. The patient underwent resection of peritoneal mass. We slowly separated the mass form front to back, from top to bottom and from left to right, the lesion was removed completely. The surgical specimen was shown in Figure 2. After wound hemostasis, each layer was sutured and incision was closed. The operation lasted 1.5 h with a total blood loss of ∼100 mL, and no transfusion was performed.

**Figure 3:**
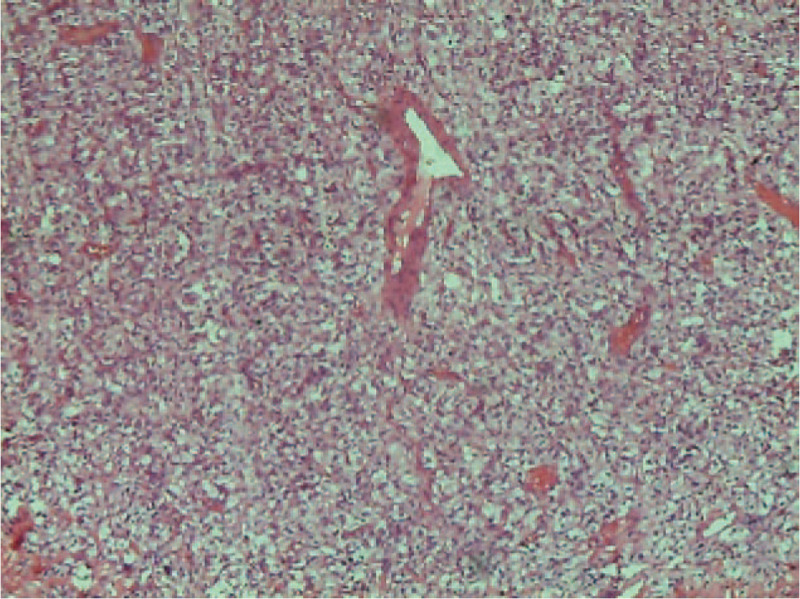
Histology and immunohistochemistry of the tumor.

According to the postoperative pathological examination, the lesion was mainly composed of chromaffin cells. Immunohistochemical staining revealed that the tumor expressed chromogranin A, NSE and synaptophysin. Based on these findings, this mass was diagnosed as benign ectopic pheochromocytoma (paraganglioma), as is shown in Figure 3.

After the surgery, arterial blood pressure was monitored continuously. In order to prevent lack of catecholamines and other substances after tumor removal, we adopted measures like administration of norepinephrine for the patient by micro-infusion pump for 5 days postoperatively, as well as 3800 mL/day of liquid therapy. When the patient's blood pressure was high, we also used nitroprusside delivered by micro-infusion pump. The patient experienced good overall recovery and was discharged 8 days after the operation. The patient left our hospital with normal blood pressure.

The patient has permitted and provided informed consent for the publication of her medical data, case details and images.

## 3 Discussion

Ectopic pheochromocytoma used to be known as the 10% tumor due notably to the observation that 10% arise at extra-adrenal sites. ^[6]^ However, most of the more recent studies undermine the 10% rule, where ectopic tumors comprise 10% to 29% of adult pheochromocytomas.^[2]^ The majority of ectopic pheochromocytomas are intra-abdominal, with the organ of Zuckerkandl being one of the most common sites.^[7]^ Named for the Austrian anatomist Emil Zuckerkandl, the organ of Zuckerkandl, is a collection of chromaffin cells spanning from the inferior mesenteric artery to the bifurcation of the aorta.^[8]^ Chromaffin cells can release a large amount of catecholamines and pheochromocytoma-associated elevated catecholamine levels can cause an increase in plasma renin activity levels, which lead to a series of symptoms.^[9]^ The most common presentations of EP include hypertension, palpitations, dizziness and headache, but there are about 24% to 75% of asymptomatic patients.^[10]^

Missed diagnosis and misdiagnosis of ectopic pheochromocytoma can happen easily. Patients mostly seek medical advice on account of visceral compression or finding of abdominal mass, presented with nonspecific symptoms described as one or more of following: palpitation, sweating, headache, dizziness, fainting, and flushing.^[2]^ For physical examination, it is helpful to monitor the patient's blood pressure when pressing the mass. Usually, when pressure on the mass causes fluctuation of blood pressure, it should alert physicians that the mass is likely to be ectopic pheochromocytoma. For biochemical diagnosis, sensitivities of plasma-free catecholamines (99%) and urinary methoxylated derivatives (97%) were the highest among biochemical tests for diagnosing pheochromocytomas.^[11]^ Should additional biochemical testing be necessary, the possibility of false-positive results due to medications, clinical conditions, or inadequate sampling conditions (e.g., blood sampling while seated) should first be considered and eliminated. In patients with plasma metanephrine values above the upper reference limit and less than 4-fold above that limit, the clonidine suppression test combined with measurements of plasma catecholamines and normetanephrine may prove useful.^[12]^ For imaging diagnosis, the sensitivity of CT, magnetic resonance imaging (MRI) and metaiodobenzylguanidine (MIBG) scintigraphy was 90%, 93%, and 91%, and the specificity was 93%, 93%, and 100%, respectively.^[13]^ Either CT or MRI is recommended for initial tumor localization, with MRI preferred in children and pregnant or lactating women due to concerns regarding radiation exposure. The specificity of scintigraphy using iodine 123 or 131 labeled MIBG is close to 100% in terms of characterizing a mass detected by CT or MRI. In the initial assessment, it is also performed to detect multifocal lesions or secondary locations of a malignant lesion. For genetic study, ectopic pheochromocytoma is currently associated with germline and/or somatic mutations in more than 20 genes. These mutations are divided into three main clusters (Pseudohypoxic signaling cluster, Kinase signaling cluster and Wnt signaling cluster) based on the activation of a particular signaling pathway and each cluster is associated with unique clinical characteristics of patients with these tumors.^[14]^

In this case, it can be revealing for us that taking full advantage of image diagnosis characteristics of ectopic pheochromocytoma helps us to avoid missed diagnosis or misdiagnosis as well as improve the accuracy of preoperative diagnosis.

The patient was admitted to our hospital mainly because of upper abdominal discomfort. Medical history included type-2 diabetes but no classical symptoms of ectopic pheochromocytoma was identified such as palpitations, headaches, profuse sweating. And during the preoperative examination of CT images, clinicians and imaging experts mainly focused on the location and size of the mass, lymph nodes, and other aspects pertinent to pancreas cancer and overlooked other possibilities. Therefore, the patient was not preoperatively diagnosed with ectopic pheochromocytoma.

As reported, the mortality was 0% to 3% for patients with adequate preoperative preparation; however, the mortality rate would go up to 43% for patients without diagnosed quiescent pheochromocytoma. Therefore, the preoperative diagnosis is one of the prerequisites for reducing the risk of perioperative anesthesia. After making the definite diagnosis, preoperative preparation steps like easing blood pressure, expansion of blood volume are quite important, which even determine to the postoperative outcome of pheochromocytoma a certain extent.^[15]^ After surgery, most patients present hypotension, and in severe cases, this may lead to cardiac arrest.^[16]^ Since our preoperative diagnosis of the patient was not ectopic pheochromocytoma, we proceeded the following measures: During surgery, we slowly separated the mass, once the patient presented hypertension, we suspended the surgery until the patient's blood pressure returned to normal levels. As soon as the surgery was done, we continuously monitored arterial blood pressure and delivering vasoactive drugs or anti-hypersensitive drugs by micro-infusion pump was performed immediately. The patient was discharged with normal blood pressure.

If preoperative preparation is done well, ectopic pheochromocytoma has satisfactory prognosis. All in all, with the extension of human life and the increasing level of medical treatment, the detection and cure rate of ectopic pheochromocytoma are set to grow further. Early and accurate diagnosis of ectopic pheochromocytoma are crucial to patients. Hence, physicians and radiologists should explore all clinical possibilities, preoperative full body work-up and imaging examination should be used well to improve diagnostic accuracy. Plus, the management of the patient's blood pressure requires comprehensive planning pre-, during, and after surgery, which can raise curative ratio.

## Author contributions

**Conceptualization:** Bing Cai.

**Data curation:** Chenshan Jiang, Jianguo Zhao, Li Sun.

**Funding acquisition:** Bing Cai.

**Writing – original draft:** Chenshan Jiang, Jianguo Zhao.

**Writing – review & editing:** Chenshan Jiang, Jianguo Zhao, Li Sun, Bing Cai.
